# E4orf1: The triple agent of adenovirus – Unraveling its roles in oncogenesis, infectious obesity and immune responses in virus replication and vector therapy

**DOI:** 10.1016/j.tvr.2024.200277

**Published:** 2024-02-28

**Authors:** Lilian Göttig, Sabrina Schreiner

**Affiliations:** a Institute of Virology, School of Medicine, Technical University of Munich, Germany; b Institute of Virology, Hannover Medical School, Hannover, Germany; c Cluster of Excellence RESIST (Resolving Infection Susceptibility; EXC 2155), Hannover, Germany; d Institute of Virology, Medical Center - University of Freiburg, Freiburg, Germany

**Keywords:** HAdV-C5, HAdV-D9, HAdV-D36, E4orf1, PI3K-Akt, AdV vectors, Oncolytic AdV, Oncogenesis, Adipogenesis

## Abstract

Human Adenoviruses (HAdV) are nearly ubiquitous pathogens comprising numerous sub-types that infect various tissues and organs. Among many encoded proteins that facilitate viral replication and subversion of host cellular processes, the viral E4orf1 protein has emerged as an intriguing yet under-investigated player in the complex interplay between the virus and its host. E4orf1 has gained attention as a metabolism activator and oncogenic agent, while recent research is showing that E4orf1 may play a more important role in modulating cellular pathways such as PI3K-Akt-mTOR, Ras, the immune response and further HAdV replication stages than previously anticipated.

In this review, we aim to explore the structure, molecular mechanisms, and biological functions of E4orf1, shedding light on its potentially multifaceted roles during HAdV infection, including metabolic diseases and oncogenesis. Furthermore, we discuss the role of functional E4orf1 in biotechnological applications such as Adenovirus (AdV) vaccine vectors and oncolytic AdV. By dissecting the intricate relationships between HAdV types and E4orf1 proteins, this review provides valuable insights into viral pathogenesis and points to promising areas of future research.

## 1 Introduction

Human Adenoviruses (HAdV) have long been recognized as causative agents of various diseases, ranging from mild respiratory infections in general, to severe respiratory, ocular, genitourinary, gastrointestinal, and neurological disorders that can be fatal in immunocompromised individuals [[1], [2], [3], [4], [5]]. To date, HAdV, non-enveloped, fiber-carrying, icosahedral viruses encapsidating approximately 36 kb of double stranded (ds)DNA, comprise more than 90 types that are quite prevalent in the human population. They are sub-typed into seven groups, A-G, according to their phylogeny, genome organization, host range, immunological cross-neutralization, recombination ability, the number of virus-associated (VA) RNA genes, hemagglutination properties, and viral oncogenic potential in rodents [[6], [7], [8], [9], [10]]. HAdV encode around 40 viral proteins that either determine the virion structure or manipulate host cellular processes to promote viral replication and evade the host's immune surveillance. Establishing a pro-viral environment is mostly ensured by the HAdV early proteins E1–4 [11]. In particular, the E4 transcript, which is multiply spliced after transcription, produces proteins necessary for a productive infection, and among these, the peptide of the first reading frame, E4orf1, has emerged in recent years as a multifunctional protein worth studying [[12], [13], [14]].

First sequenced and compared to HAdV-C2 in 1992, E4orf1 of HAdV-C5 (E4orf1-C5) was considered a protein with non-essential function, since its deletion resulted in unaltered growth kinetics in viral plaque assays in HeLa cells [12,14,15]. However, two years later Javier published a ground-breaking finding, showing that previously observed estrogen-dependent mammary tumors in rats originating from HAdV-D9 infections were dependent on E4orf1 (E4orf1-D9) expression [16,17]. Around the same time another HAdV, -D36, was being investigated for its adipogenic potential in animal models and human tissue, but it was not until 2008 when E4orf1 (E4orf1-D36) was identified as the causative agent for this pro-metabolic action [[18], [19], [20], [21], [22], [23]], as recently reviewed in Hernandez-Magaña et al., 2023 [24]. Since then, HAdV-D9 and -D36 have been extensively studied, while only recently, investigation of E4orf1-C5 is proving it to be more “essential” than previously thought. For instance, novel research is showing that E4orf1 may be an essential factor regulating host immune responses in Adenovirus (AdV) vectors [25].

Thus, understanding the role and functions of E4orf1 in the context of these viral types is crucial for unraveling the intricate interplay between the virus and its host, shedding light on the mechanisms underlying viral replication and pathogenesis. In this review, we aim to provide a comprehensive overview of E4orf1, focusing our description on HAdV-D9, -D36, and -C5, briefly discussing its expression in the viral life cycle and its structure, as well as highlighting its functional properties during HAdV infections and overexpression. Furthermore, we discuss recent biotechnological findings that offer exciting yet possibly precarious prospects for developing novel and innovative AdV vector systems.

## 2 HAdV infection lifecycle and the expression of E4orf1

The life cycle of HAdV encompasses a series of well-coordinated steps involving viral attachment, entry, genome replication, gene expression, virion assembly, and release. Throughout this intricate process, the viral proteins play crucial roles in manipulating the host cellular machinery to ensure efficient replication and dissemination of the virus. Commonly, HAdV undergo a lytic replication cycle, where they infect different tissues, including the epithelium of the respiratory and gastrointestinal tract, the ocular conjunctiva, the urinary tract, and more rarely the central nervous system [26]. Since the adenoviral entry and early replication steps have been comprehensively reviewed before [[27], [28], [29]], we will not discuss the initial HAdV infection steps here.

**Fig. 1:**
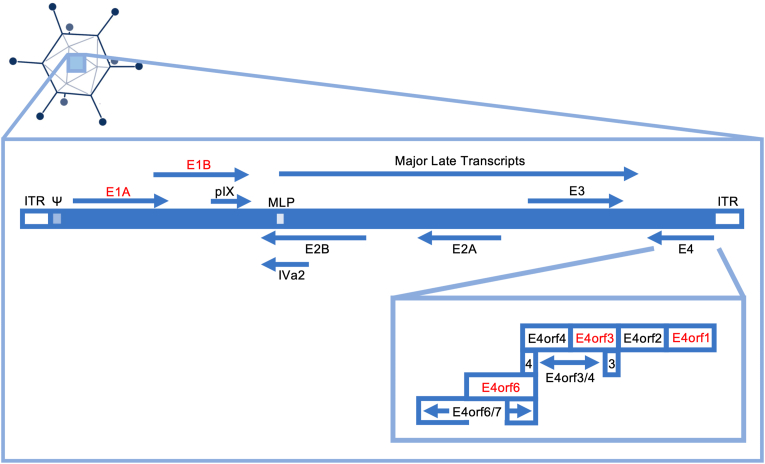
**HAdV genome organization**. The viral DNA genome is shown as a blue box of around 36 kb with the ITRs at either end, packaging sequence Ψ, and MLP (Major Late Promoter). The arrows indicate the orientation of the initial transcription units, E1-4, pIX, IVa2, and Major Late Transcripts. The E4 region is expanded to show the splice products: E4orf1, E4orf2, E4orf3, E4orf4, E4orf3/4, E4orf6, E4orf6/7. The proteins marked in red represent HAdV oncogenes.

Upon nuclear localization of the HAdV genome, E1A is the first protein to be transcribed and expressed, which then acts as viral transactivator, initiating the transcription of the other HAdV genes [30,31]. The far right of the HAdV genome, transcribed in the opposite direction, encodes the E4 region, which is transactivated by E1A from a single promoter shortly after infection, with expression persisting throughout the late phase of infection [32,33]. The E4 region encodes one complete mRNA precursor molecule, which is diversely spliced to produce at least six functional peptides (Fig. 1) [[34], [35], [36]]. Although HAdV mRNA splicing plays a central role in protein expression, how many, and what relative abundance of viral ORFs are expressed at what time of infection is only beginning to be resolved [34,35]. In HAdV-C5, novel research indicates that the synthesized E4 mRNAs include predominantly early class spliced mRNAs, such as E4orf2, E4orf3, E4orf4 and E4orf6, which peak at 12 hpi, and mRNAs of the later class, E4orf1 and E4orf6/7, which are most abundantly at 24 hpi, although interestingly, E4orf1 is the first E4 region to be transcribed (Fig. 1) [37].

Closely involved in DNA replication, late gene expression, and host protein synthesis shut-off, the E4 proteins and their functions during infection are variously regulated through post-translational modifications (PTM), interactions with other HAdV and/or cellular proteins, and spatio-temporal expression [14,[38], [39], [40], [41]]. Since the functions of the E4 proteins diversely regulate transcription, cell cycle progression, apoptosis, DNA repair, cell signaling, PTMs, and the integrity of the cellular antiviral PML nuclear bodies (PML-NB) [[42], [43], [44]], deletion of the entire E4 region leads to a significant reduction in viral replication, yet deletion of one single ORF of E4 has only small effects [14,41]. Together with the E1 proteins, some E4 proteins have partial transforming capacity and in the correct setting, so does E4orf1 alone [17,42,45,46]. Later during infection, E4 transcription is diminished through inhibitory E2A and negative feedback repression mediated by the E4orf4 protein [47,48].

## 3 Host cell transformation by HAdV oncogenes

Although HAdV are not linked to cancer development in humans to date, certain subgroups have been shown to cause oncogenic transformation in rodents and primary cells. In 1962, initial research showed that HAdV-A12 can induce tumors in new-born hamsters, which since then classified HAdV as DNA tumor viruses [49]. Today, HAdV-A12, A18, and A31 are known to be the most oncogenic HAdV, while some HAdV of species D and B are also comparably oncogenic in rodents [50,51]. Nevertheless, primary rodent cells with diminished cellular immunity can be transformed by most if not all types of HAdV A-G, and rodent cells transformed by non-oncogenic HAdV can produce tumors when grafted onto immunosuppressed animals [[50], [51], [52]]. Furthermore, a handful of primary human cells have been transformed by HAdV to generate cell culture systems, including human embryonic kidney cells, human embryonic retinoblasts, human mesenchymal stromal cells, and amniocytes [[53], [54], [55], [56], [57]]. In humans, the presence of HAdV DNA has been found in pediatric brain tumors, small-cell lung carcinomas, mantle cell lymphomas, and human sarcomas [[58], [59], [60], [61]]. However, a causative link to HAdV-induced cancers in humans has never been shown so far [42,52].

Transformation by HAdV does not result in a productive infection, since only a certain set of genes are expressed; these are not able to produce virions but are sufficient to cause malignant changes in the infected cell. During HAdV transformation, the viral oncogenes may persist in the transformed cells and can be constitutively expressed. Initially, E1A and the E1B proteins E1B–55 K or E1B–19K, act in concert, where E1A first modulates cellular gene expression to drive cell proliferation and immortalization [50,62,63]. Complete transformation is achieved by mainly E1B–55K or E1B–19K that block apoptotic pathways of the cell [[64], [65], [66], [67]]. The success of E1B–55K-mediated transformation depends on the interaction with the tumor suppressor protein p53, inhibition of p53-mediated transcription, and the nuclear export of p53 [68,69]. Also, E4orf3 and E4orf6 individually contribute towards transformation by HAdV. For instance, E4orf3 reorganizes key antiviral protein aggregates, including the PML-NB and the MRN complex, supporting cell proliferation and interfering with cellular double strand break repair, respectively, while E4orf6 inactivates among others p53 and its transcriptional activity [42,[70], [71], [72], [73], [74], [75]]. Moreover, one of the key oncogenic mechanisms is the degradation of p53 by the HAdV-E3 ubiquitin ligase complex comprising E4orf6 and E1B–55K [76,77]. With E1A and E4orf3 and/or E4orf6, but without E1B–55K, primary baby rat kidney cells can be transformed, often losing viral oncogene expression, indicating that transformation occurs through a hit-and-run mechanism [78]. Since these viral proteins seem to be the causative agents, the hypothesis remains that even if tumors lack virus-specific molecules, a viral origin may be more prevalent than currently recognized [78]. As discussed in greater detail below, and recently reviewed [24], E4orf1 has now been ranked as an HAdV oncogene, but transforming capabilities together with HAdV-C5 E1A or the E1B proteins have so far not been explored and remain an intriguing area of investigation.

## 4 Nucleotide composition and protein structure of E4orf1

**Fig. 2:**
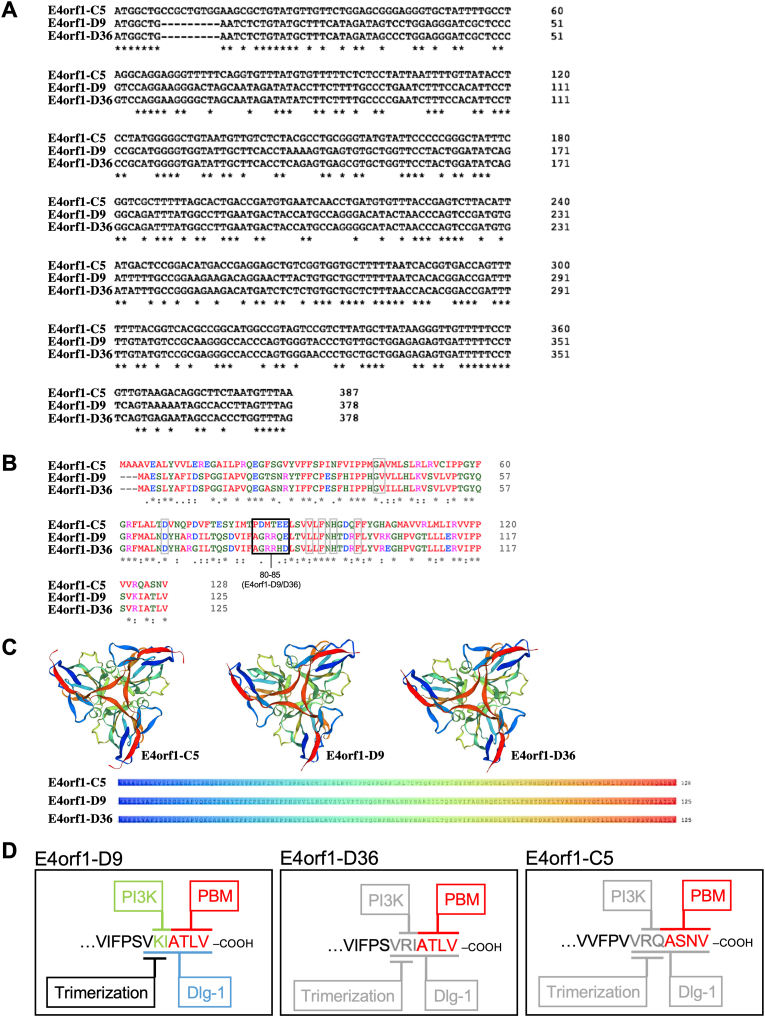
**E4orf1 sequence analysis**. (**A**) Multiple gene sequence alignment of E4orf1-C5, -D9, and -D36 with Clustal Omega (EMBL-EBI; [346]). (**B**) Multiple protein sequence alignment of E4orf1-C5, -D9, and -D36 with Clustal Omega (EMBL-EBI; [346]). “*” indicates conservative residues in all three sequences, “:” represents residues with strongly similar properties, and “.” represents residues with weakly similar properties. (**C**) *In silico* prediction of E4orf1-C5, -D9, and -D36 protein tertiary structures based on SWISS-MODEL Workspace computation [88,89] with sequence coloring highlighting the strand folds within the tertiary structure [88]. (**D**) E4orf1 carboxy-terminal domain of E4orf1-D9, -D36, and -C5 with the known PBM domain of all three types (red), as well as the known PI3K-interacting domain (green), Dlg1-binding domain (blue), and trimerization domain (black) for E4orf1-D9. Putative but unresolved PI3K-interacting, Dlg1-binding, and trimerization domains for E4orf1-D36 and -C5 are indicated in grey.

The E4orf1 DNA sequence demonstrates a considerable degree of conservation across HAdV species, although HAdV-F40 and F41 have lost the coding sequence for E4orf1, suggesting possible functional redundancy met by another HAdV-F protein [79]. *In silico* analysis shows that the HAdV-C5 E4orf1 gene is approximately 49% conserved with HAdV-D9 and -D36, while the latter two are 93% conserved within their nucleotide sequences (blastn; [80]). HAdV-D9 and -D36 E4orf1 genes have a deletion of nucleotides 8–16 compared to HAdV-C5 E4orf1 (Fig. 2A).

The primary peptide sequence of E4orf1 varies among different HAdV types, but it typically comprises approximately 120–130 amino acids [81]. Despite sequence variations, E4orf1 proteins share certain conserved motifs and functional domains that contribute to its functions [82,83]. Analysis from basic alignment tools can identify conserved peptide regions and out of 125/128 amino acids, 53 (∼42.5%; Fig. 2B “*”) are conserved equally throughout the peptide sequences, 25 (∼20%; Fig. 2B “:”) are conservative with similar properties mainly in the middle and end of the peptides, and 12 (∼9.5%; Fig. 2B “.”) are conservative with weakly similar properties primarily at the beginning of the peptide sequences. These alignments also reveal that overall homology for physiochemical amino acid assembly is highly conserved between E4orf1 HAdV-D9 and HAdV-D36, and to a lesser extent between all three peptides. Global analysis of the peptide sequence indicates that while E4orf1-C5 is approximately 45% conserved with HAdV-D9 and -D36, it is 62–63% conserved when considering conservative amino acid substitutions (blastn; [80]). HAdV-D9 and -D36 share the same peptide length and are 92% (conservative identities) or 96% (positive identities with conservative substitutions) similar (Fig. 2B).

Evidence suggests that HAdV E4orf1 evolved from a eukaryotic dUTP pyrophosphatase (dUTPase), which usually prevents harmful incorporation of uracils into replicating cellular DNA by hydrolyzation [84,85]. E4orf1 of some if not all E4orf1-containing HAdV are similar in length and usually have a conserved protein shape. Although E4orf1 proteins can structurally form homotrimers like their ancestor proteins, cellular dUTPases, the viral proteins have functionally diverged from the cellular enzymes, and, contrary to dUTPases, also exist as monomers [84,86]. Predicted to express as 14.3 kDa (E4orf1-C5) and 14 kDa (E4orf1-D36) (uniprot.org; [87]), the tertiary protein structure may form homotrimers (SWISS-MODEL [88,89]; license https://creativecommons.org/licenses/by-sa/4.0/legalcode) (Fig. 2C). Based on the crystal structure of human dUTPase, a β-strand 1 (β1) from one subunit and C-terminal β-strand 8 (β8) from an adjacent subunit are predicted to mediate the intermolecular interaction that stabilizes trimer formation [86,90]. Experimentally, the homotrimeric assembly of E4orf1-D9 was elucidated to occur through the residues VKI located at the C-terminus (Fig. 2D) [45,91,92]. Using size exclusion chromatography and comparing elution with human dUTPase, requirement for the β8-residues was confirmed in E4orf1-D9 trimer formation. Mutational analysis of E4orf1-D9 revealed that the C-terminal, PBM-adjacent residues (see Section 5), VKI, contribute to homotrimerization: mutations to AAI and VAA still eluted as trimers, whereas AKA eluted as a mixture of trimers and monomers, reflecting some kind of defect in trimer formation, and AAA eluted as a monomer, which also failed to self-bind in immunoprecipitation assays. Thus, these results defined the β8-residues VKI as a crucial E4orf1-D9 trimerization element [86]. Intriguingly, E4orf1-D36 has VRI and E4orf1-C5 has VRQ residues (Fig. 2D).

E4orf1-D36 is the only D type that has the lysine substituted to an arginine in the trimerization domain, posing the question of whether the peptide exists preferentially as a homotrimer or as a mixture of trimers and monomers [93,94]. Although efforts have been made to elucidate the protein folding of E4orf1-D36 with interaction partners *in silico*, the homotrimer structure was not determined in the study, also due to limitations posed in the computational analyses [93]. No models exist so far for E4orf1-C5, however differences in molecular weight in Western blots have been reported [95](Göttig et al., JVI manuscript accepted). Therefore, although E4orf1 homotrimerization is predicted, it is important to note that the computational models are based on predictions and require experimental validation as performed for E4orf1-D9. Experimental techniques with mutational analyses, X-ray crystallography, or NMR would provide detailed structural information about E4orf1. Furthermore, both the global landscape and individual PTM sites on E4orf1 are so far unknown and experimentally untested.

## 5 .Functional domains of E4orf1, particularly the PBM

Conserved throughout the HAdV species containing E4orf1, the best-described functional domain within E4orf1 is the hydrophobic carboxy-terminal PBM [9,91]. The PDZ (Postsynaptic Density (PSD95), Discs Large (Dlg), and Zonula Occludens (ZO-1) -binding cluster) binding motif (PBM) recognizes PDZ protein-protein recognition sequences that are usually approximately 80–90 amino acids long [96,97]. PDZ proteins are often present at the plasma membrane, acting as scaffolds, and no less than 400 distinct proteins with PDZ domains exist in humans [98,99]. The functions of these proteins include organizing signaling complexes at neurological synapses, contributing to fundamental processes of the cellular immune response, forming cell-cell junctions, supporting cell proliferation and survival, as well as intracellular trafficking, and apico-basal polarity [[100], [101], [102], [103]]. Depending on the cellular environment, deregulated PDZ proteins may act as oncogenes or tumor suppressors, contributing to tumorigenesis, where their relative abundance and sub-cellular localization drive their function [[103], [104], [105]].

Carboxy-terminal PBMs usually occur in three classes: class I –[X–S/T–X–ΦCOOH], class II –[X–Φ–X–ΦCOOH], and class III –[X–D/E–X–ΦCOOH], where Φ is a hydrophobic residue and X is any residue [[106], [107], [108]]. As class I PBMs, E4orf1-D9 and -D36 contain -ATLV as their PBM, and E4orf1-C5 has -ASNV (Fig. 2D). Besides E4orf1, viral PBM proteins include HTLV (human T-lymphotropic virus type 1) Tax-1, high-risk HPV (human papilloma virus) E6, and HBV (hepatitis B virus) Core (although with an atypical PBM featuring a cysteine instead of S/T/Φ/D/E); they can modulate the functions of PDZ proteins to support viral replication, spread, and pathogenesis [[109], [110], [111], [112], [113]]. For HPV E6 proteins, the oncogenic potential may directly correlate with a broader capacity to interact with multiple PDZ proteins [114]. Interestingly, although E4orf1 sequences in recently identified circulating strains of HAdV-41C1 and -1C2 have undergone multiple amino acid substitutions, the carboxy-terminal regions retain conserved and functional PBMs [9].

The molecular mode of action of the PBM has been best described for E4orf1-D9. Specifically, E4orf1-D9 binds the PDZ proteins MUPP1, MAGI-1, ZO-2 and PATJ, thus disrupting tight junction barriers and apicobasal polarity, which contributes to the cancerous phenotype of E4orf1-modulated cells [91,[115], [116], [117], [118], [119], [120]]. Monomeric E4orf1-D9 preferentially binds the PDZ proteins above, and as a trimer it mostly sequesters the PDZ protein Dlg1 [86]. E4orf1-C5, -D9, and -D36 all interact with Dlg-1, which ultimately mediates activation of the host phosphatidylinositol 3-kinase (PI3K; see Section 6); however, whether this involves trimerization of the E4orf1-C5 and -D36 proteins has not been validated experimentally [95]. Intriguingly, Dlg1 is also bound by most if not all of the HPV PBM-containing E6 proteins and at least MUPP1 and MAGI-1 are known to interact with high-risk HPV E6, which may indicate an evolutionarily conserved interaction apparently mirrored by HAdV E4orf1 [95,114,118,119]. In contrast, ZO-2 fails to interact with E4orf1-C5, indicating that the cooperation between the above named PDZ proteins and the E4orf1 types does not occur equally for all, and that exclusive PDZ interactions with E4orf1-D9 may account for its more potent transforming and tumorigenic properties [91,120].

The primary peptide sequence of E4orf1 is generally grouped into three semi-conserved domains, with an amino-terminal domain 1, a central domain 2, and a carboxy-terminal domain 3/PBM, designated originally by the transforming capacity of E4orf1-D9 mutants in rat embryo fibroblast cells (CREF), although the functional significances of the domains are not yet fully understood [45,46]. However, domain 2 seems to play a pivotal role in mediating PI3K-dependent transformation in E4orf1-D9, since at least residues L89, F91, F97, G40, D65, and H93A mediate PBM-independent cellular transforming activity, while E4orf1-D9/36 residues G40, V41, D65, L89, F91, H93, and F97 are thought to mostly define domain 2, although V41 and L89 are not conserved in E4orf1-C5 (Fig. 2B, grey boxes) [46,121]. A recent *in silico* modeling study of E4orf1-D36 characterized its tertiary peptide structure and analyzed its interactions with PDZ domain-containing proteins (PDZ-1, 2, 3 7, and 10) using homology modeling, docking, and molecular dynamics. In particular, the E4orf1-D36 interaction with PDZ10 was identified as a highly stable complex, which depends not only on the PBM of E4orf1 but also on residues 80–85 in domain 2, which contributed to longer stabilization of the complex [93]. Interestingly, peptides 80–85 are not highly conserved between E4orf1-C5 and D36, but more so between E4orf1-D9 and D36 (80–83 highly conserved; Fig. 2B, black box).

In summary, the structure and molecular properties of E4orf1 play a crucial role in its multifaceted functions during HAdV infections. The conserved domains and conformational dynamics of E4orf1 contribute to its interactions with host factors and modulation of cellular processes. Further studies aimed at elucidating the high-resolution structure of E4orf1 to help unravel its molecular mechanisms will enhance our understanding of its functions and potential biotechnological applications.

## 6 Consequences of interfering with the cellular PI3K-Akt-mTOR pathway

**Fig. 3:**
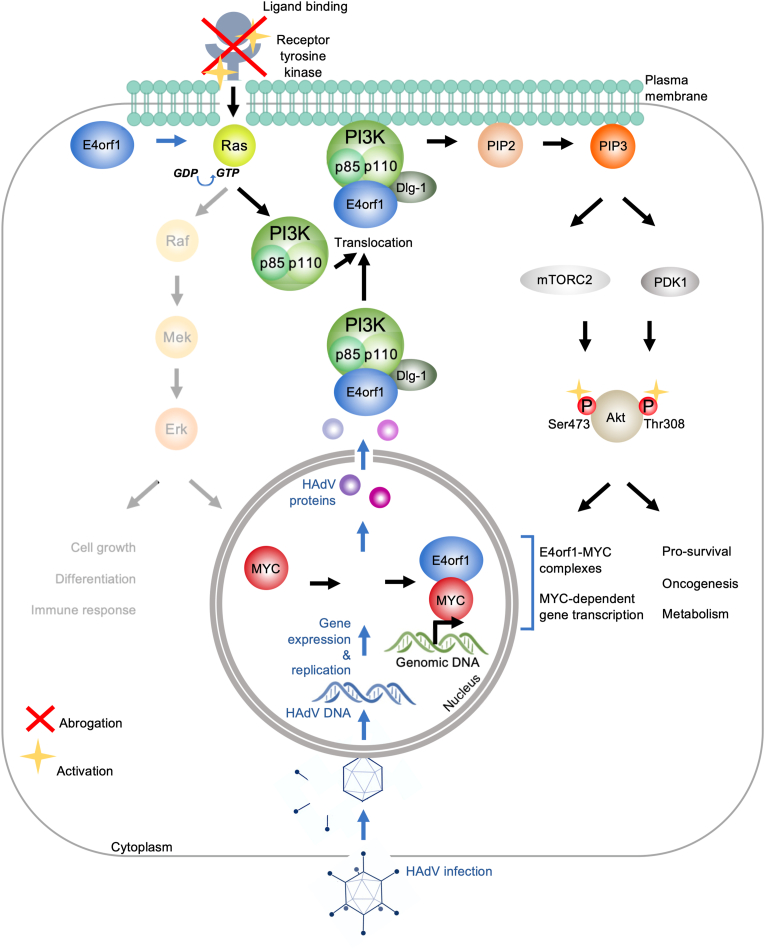
**Overview of the E4orf1 molecular mode of action**. Schematic overview of various interactions between E4orf1 (blue) and cellular proteins, including the consequences of such interactions. See the main text for detailed descriptions that refer to this figure. HAdV infection and HAdV-induced mechanisms are described in blue, including arrows, while cellular mechanisms are described in black, including arrows. The Raf-Mek-Erk signaling pathway, which induces MYC expression, is faded out, since although HAdV infection activates this pathway, the E4orf1 mode of action is suggested to strongly favor PI3K-Akt-mTOR pathway activation. Red crosses are abrogating modes of action, and yellow stars represent activating signaling.

Primarily, initial receptor signals are transmitted from the cell surface to intracellular pathways by the Ras proto-oncogenes, a group of small GTPases widely mediating cell proliferation, differentiation, migration, and apoptosis (Fig. 3; [122]). Ras signaling is activated by various cellular receptors, including receptor tyrosin kinases (RTKs), G-protein coupled receptors, cytokine receptors, and extracellular matrix receptors. Ras proteins act as molecular switches, cycling between an active GTP-bound state and an inactive GDP-bound state. This exchange is controlled by guanine-nucleotide-exchange factors (GEFs) that activate Ras by promoting GDP-GTP exchange, and GTPase-activating proteins (GAPs) that stimulate GTP hydrolysis and inactivate Ras [123]. Once activated, Ras-GTP interacts with downstream effectors including mitogen-activated protein kinases (MAPK) and PI3K pathways, while the best-described Ras-effector is the Raf-MEK-ERK signaling axis, which also regulates cell growth, differentiation, inflammation, and apoptosis [124,125]. Within the latter pathway, a MAPKKK protein (Raf) acts as the initial GTPase that phosphorylates and activates an intermediate kinase, MAPKK (MEK), which in turn phosphorylates and activates the effector kinase MAPK (ERK; extracellular signal-regulated kinase) (Fig. 3; 124).

(Ras-)Raf-MEK-ERK and (Ras-)PI3K-Akt-mTOR (mammalian/mechanistic target of rapamycin) pathways are tightly integrated, since they can induce negative feedback loops, cross-inhibition, cross-activation, or pathway convergence, the latter for instance regulating cellular MYC activation. Molecularly, the direct interaction between GTP-bound Ras and PI3K elevates the activity of PI3K, either boosting its catalytic activity or promoting tighter interaction with the plasma membrane [[125], [126], [127], [128]]. The strength and duration of pathway activations are regulated by the reciprocal feedback loop and the intensity of the stimulus. Since the agonists activating the Raf-MEK-ERK and the PI3K-Akt-mTOR pathway are mostly distinct, where for instance phorbol-12-myristate-13-acetate is a potent activator of Raf-MEK-ERK, while insulin and insulin-like growth factor 1 (IGF1) are weaker activators of Raf-MEK-ERK but strong activators of PI3K-Akt-mTOR [126,129,130]. However, the extent of pathway activation by specific growth factors often depends on determinants such as the concentration of the growth factor, the expression and localization of the corresponding RTK, and the presence of receptor family members and auxiliary proteins [131]. For instance, inhibiting PI3K results in E4orf1 strongly activating the Raf-MEK-ERK pathway, which seems to be conserved between HAdV types, ultimately also supporting the late-phase of infection [[132], [133], [134]].

The epidermal growth factor receptor (EGFR), the insulin receptor (IR) and the related IGF1 receptor (IGF1R) represent well-studied and specialized forms of RTKs and the receptor-mediated signaling cascade that activates PI3K-Akt-mTOR. As transmembrane proteins with an intracellular tyrosine kinase domain and extracellular ligand binding sites, EGFR is activated and dimerizes upon EGF protein binding, while insulin or IGF1 and IGF2 bind and activate homo- and heteromeric IR/IGF1R complexes [135,136]. The extracellular ligands are EGF family proteins for EGFR, and insulin or IGF1 and IGF2 for InsR/IGF1R. These convey conformational changes, tyrosine kinase activity and tyrosine autophosphorylation in the cytoplasm, which can then recruit effector proteins of signaling cascades, including the Ras-Raf-MEK-ERK and PI3K-Akt-mTOR pathways, while cross talk between EGFR and IR/IGF1R can also occur [[137], [138], [139]].

The canonical PI3K-Akt-mTOR pathway, a central cellular regulatory route, is meticulously controlled through spatial and temporal compartmentalization at the inner plasma membrane (Fig. 3). Activation from ligand binding, G protein-coupled receptors or receptor tyrosine kinases, triggers class IA PI3Ks, consisting of p110α/β/δ and a regulatory subunit (p85α/β, p55α/γ, or p50α) [140]. Active PI3K converts phosphoinositol-lipid PIP2 to PIP3, recruiting and activating Akt isoforms 1–3 through phosphorylation [141,142]. While Akt1 and 2 are widely expressed, Akt2 is pivotal in metabolic tissues, and Akt3 in the brain [143]. PDK1 phosphorylates Akt1 Thr308, activating mTORC1 for protein synthesis, and Akt1 Ser473 phosphorylation by mTORC2 or DNA-PK completes its activation (Akt2, Thr309 and Ser474; Akt3, Thr305 and Ser472) [[144], [145], [146], [147], [148], [149]]. With over a hundred Akt substrates, the numerous phosphorylation cascades trigger many processes such as cell cycling, angiogenesis, and growth, while also inhibiting apoptosis (Fig. 3) [[150], [151], [152], [153], [154], [155]]. The Akt-Thr308 and -Ser473 residues are dephosphorylated by PP2A and PHLPP1/2, respectively, thus inactivating Akt, while suppression of PIP3 by PTEN abrogates upstream Akt signaling, suppressing for instance growth and survival [[156], [157], [158]].

Since full activation of Akt spurs both cytoplasmic and nuclear cascades, Akt downstream action can include the inhibition of apoptotic factors (FoxO), the phosphorylation of Mdm2 to modulate p53-mediated apoptosis, and the regulation of cell cycling by affecting p21 and p27 [[159], [160], [161], [162], [163], [164], [165], [166]]. Furthermore, active Akt modulates glucose and lipid pathways, as well as insulin-driven glucose transport. Specifically, insulin activates IR, which leads to Akt phosphorylating TBC1D1, ultimately translocating glucose receptors (GLUTs) [167]. To complement insulin-directed glucose metabolism, Akt inhibits FoxO1 leading to gene expression for gluconeogenesis and fatty acid oxidation, represses GSK-3 for energy regulation [[168], [169], [170], [171], [172], [173]], phosphorylates and activates glycolytic enzymes (HK2, PFKFB2), and promotes expression of the glucose transporters and glycolytic enzymes though PI3K-Akt activation of MYC [[171], [172], [173]]. Additionally, Akt can activate cholesterol and lipid synthesis, for instance through SREBP induction [174]. Due to the broad activity spectrum of the PI3K-Akt-mTOR pathway, disruptions at any point in the pathway contribute to diseases such as cancer and diabetes [154]. Faulty receptors may either hinder PI3K-Akt signaling despite ligand binding (Diabetes Mellitus 2) or covey over-activating signals (EGFR-positive cancers) [[175], [176], [177], [178]]. Also, mutations in PI3K or Akt, or suppressor malfunctions (PTEN, TSC2), can over-activate mTORC1, ultimately also known to cause cancer [127,[179], [180], [181], [182], [183], [184], [185], [186], [187], [188]]. Lastly, defective downstream factors can disrupt apoptosis (Mdm2-p53), survival (FoxO, Bcl2), and overall lipid and glucose metabolism (SREBPs, GLUT, GSK-3, MYC, etc.) [142,[189], [190], [191]].

Early research revealed that E4orf1-D9 indeed stimulated the PI3K-Akt-mTOR pathway by a Ras-dependent mechanism that failed to equally activate the Raf-MAP-ERK pathway, leading to cell-cycle progression marked by p70^S6K^, and ultimately transformed CREF cells, which was counteracted by the PI3K inhibitors wortmannin, LY294002, and rapamycin, which specifically inhibits mTOR and downstream p70^S6K^ [121,132]. Moreover, PI3K-Akt-mTOR activation through E4orf1 occurs through direct interaction with class I A PI3Ks, enhancing cell survival and influencing metabolism in the host cell, ultimately also promoting HAdV replication (Fig. 3) [121]. E4orf1 achieves PI3K activation by recruiting the PDZ protein Dlg1 through its PBM and binding the regulatory and catalytic subunits of PI3K dependent on Dlg1 interaction. This trimeric complex forms at the plasma membrane and activates downstream Akt [95]. For E4orf1-D9 and -C5, mutations in the PBM abrogate Akt activation, and mutant E4orf1-D9 fails to induce transformation of human epithelial cells, as is achieved by the wild type (wt) protein [92] (Göttig et al., JVI manuscript accepted). Concomitantly, E4orf1 from certain HAdV subtypes of A-D were shown to also activate PI3K in a Dlg1-dependent manner [95,110]. Furthermore, mutational analysis of E4orf1-D9 revealed that residues 89–91 within domain 2 can also activate the PI3K, contributing to E4orf1-D9 mediated transformation, whereas the PI3K-stimulatory activity of domain 2 in E4orf1-C5 and -D36 remains to be investigated [45,46].

Thus, E4orf1 potently activates the PI3K-Akt-mTOR pathway, and as will be discussed in greater detail below, expression of E4orf1 within the correct cellular environment as well as infections from distinct HAdV types can have various outcomes, including transforming capacity and metabolism modulation.

## 7 HAdV triple agent 1: HAdV-D9 E4orf1 and its role as a viral oncogene

As the largest sub-group, HAdV-D types are the most frequent cause of mild ocular infections, which can result in highly contagious epidemic keratoconjunctivitis (EKC) [192,193]. First isolated in 1957, HAdV-D9 was found to infect human corneal epithelial cells *in vitro*, but has since then not been associated with severe ocular infections, yet may rather be linked to causing gastroenteritis [[194], [195], [196], [197]]. Although other HAdV-D types such as -D8 are more commonly detected in EKC cases, the exact relationships between conjunctivitis and oncogenic potential, as well as the role of E4orf1 between the D-types and their pathology remain intriguing areas of investigation [198,199].

As described in Sections 4, 5, early research into the functional domains, interaction partners and tertiary structure of E4orf1 was performed on E4orf1-D9 due to its significant role in oncogenic transformation through activating the PI3K-Akt-mTOR pathway, producing estrogen-dependent mammary tumors in rats and rat embryo fibroblast cells (CREFs) upon HAdV-D9 infection; requiring E4orf1-D9 expression, such transformation was shown to be independent of the oncogenic E1A and E1B regions [17]. Although E4orf1s from HAdV-A12, -B3, and -C5 were unable to induce transformation in CREFs in the same study, possibly due to an E4orf1 expression defect, the E4orf1 genes from HAdV-A to D were stably expressed in the human mammary epithelial cell line MCF10A and formed foci with varying efficiency in soft agar, a strong *in vitro* indicator that correlates with tumorigenic potential [17,82,95]. This suggests that most, if not all, HAdV E4orf1 proteins have oncogenic potential in certain human cells, raising possible safety concerns about current and future uses of E4orf1-expressing vectors. Nonetheless, E4orf1-D9 is regarded as the most oncogenic agent, which may depend on its unique capability to selectively interact with the PDZ and tumor suppressor protein ZO-2, leading to its strong localization in the cytoplasm. This enables potent PI3K-Akt-mTOR activation, since E4orf1-D9 is able to bind both p85 regulatory and p110 catalytic PI3K, as well as Dlg1 itself, highlighting that E4orf1-D9 also promotes Dlg1 oncogenic activity to direct PI3K activation [92,120,132].

Furthermore, E4orf1-D9 activates (dependent on its PBM and domain 2 residues) the Ras-MEK-ERK pathway through EGF-independent EGFR activation [134]. Through E4orf1–Dlg1 binding with EGFR, it was proposed that E4orf1-D9 interferes with the function of IR–IGF1R to downregulate the autophosphorylation of EGFR and the Ras-MEK-ERK pathway to promote a second function of the IR–IGF1R complex, the upregulation of MYC expression [134]. It is thought that E4orf1-D9 redirects the activity of IR-IGF1R to form the inhibitory complex on EGFR, where the activated IR-IGF1R can stimulate (a) so far unidentified signaling pathway(s) that contribute(s) to the constitutive expression of MYC and to establishing nuclear E4orf1–MYC complexes that may stimulate anabolic glucose metabolism and nucleotide biosynthesis for efficient HAdV-D9 replication [134,200,201]. Furthermore, the activation of the Ras-MEK-ERK pathway was shown to be conserved between HAdV species -A12, -B3, and -C5 in infection [134].

Although never thoroughly investigated for its metabolic potential, HAdV-D9 has been reported to have an adipogenic effect, which might be attributed to the 94% homology between E4orf1-D9 and -D36 (blastp [80]) [202,203]. HAdV-D9 infection of mouse 3T3-L1 preadipocytes resulted in increased intracellular lipid accumulation and enhanced differentiation through the upregulation of C/EBP-β and PPAR-γ, and decreased expression of leptin (see Section 8; 203). While infected cells showed decreased secretion of the inflammatory cytokines tumor necrosis factor (TNF) and IL-6, human individuals with anti-HAdV-D9 antibodies were reported in one study to have alterations in serum lipid levels, as well as higher body weight and BMI (body mass index) [203]. Although not molecularly resolved, the interplay between EGFR, IR, Ras, and PI3K is highly likely to contribute to adipogenesis when investigated in a suitable tissue or cell environment.

## 8 HAdV triple agent 2: HAdV-D36 E4orf1 and its role as a metabolism activator

Several metabolic processes are directly regulated by oncogenes and tumor suppressors, which is why deregulation of the PI3K-Akt-mTOR pathway is associated with metabolic disorders such as obesity and Diabetes Mellitus 2, while altering both lipid and glucose metabolism (Warburg effect, reviewed for HAdV in Ref. [204]) are increasingly regarded as hallmarks of cancer [205]. Although PI3K-Akt-mTOR signaling promotes glucose uptake and lipid synthesis as well as inhibits lipolysis and β-oxidation of fatty acids, the precise balancing of lipogenesis and glucose transport is more intricate [205]. Obesity is driven by hypertrophic adipose tissue, which is mainly formed by PI3K-Akt-mediated glucose uptake and subsequent excessive triglyceride conversion and deposition [206]. Glucose and fatty acids in the blood stimulate pancreatic β-cells to produce insulin, which is responsible for keeping lipid and glucose homeostasis between circulating blood and tissue. Increased blood glucose levels elevate insulin levels, which primarily bind to IRs in the liver, adipose tissue, and skeletal muscle, proximally activating downstream PI3K-Akt-mTOR. In adipose tissue, downstream effectors enhance lipid production while preventing lipolysis [207,208]. Insulin resistance occurs when cells do not respond to insulin as intended and are unable to take up glucose for their energy metabolism. To compensate for exacerbated glucose supplies, β-cells continuously produce excess insulin and if the state of insulin resistance persists throughout a prolonged period, insulin-sensitive cells become insulin-resistant and β-cells deteriorate [[207], [208], [209]]. Insulin resistance in adipose tissue can lead to abnormal glucose metabolism [210,211], causing adipose tissue to become dysfunctional and often enlarged, fostering a pro-inflammatory and hyperlipidemic environment that ultimately promotes adipose hypertrophy and obesity [212]. Not only do excessive adipose tissue and obesity increase the risk of susceptibility to infections, but infectious agents are also being discussed as a cause of obesity and as being more easily hosted in obese individuals [213,214].

Overall, HAdV infections in humans may be linked to obesity, since the presence of type-unspecific antibodies against HAdV can correlate with adiposity and body weight [215]. Specifically, 5 subtypes, HAdV-C5, -D9, -D31, -D36, and -D37 have been associated with increased adiposity *in vitro* or *in vivo*, with HAdV-D36 being the most prominent type [202]. First isolated in 1980 from stool from a child with diabetes and enteritis, HAdV-D36 was later shown to be causative in increasing adipose tissue in chickens, mice, and monkeys, with animal obesity being transmissible through blood from HAdV-D36 infections [18,19,23,216,217]. HAdV-D36 infections also maintain obesity and reduce weight loss, but improve glycemic control in mice because cellular glucose uptake and energy availability is improved [[218], [219], [220]].

*In vitro*, HAdV-D36 infections advance adipogenesis, increase differentiation and lipid accumulation in mouse 3T3-L1 preadipocytes and human adipose-derived stem cells (hASC), and elevate insulin sensitivity and weight gain in infected rats [20,21,221,222]. Furthermore, adipocyte-committed differentiation supports HAdV-D36 productive replication and correlates with activation of MYC-dependent target genes involved in glucose metabolism [223]. Although MYC levels were not measured in this study, the target genes included Hexokinase 2 (HK2), Phosphofructokinase 2 (PFK2), Glyceraldehyde 3-phosphate dehydrogenase (GAPDH) and Lactate dehydrogenase A (LDHA), and increased expression of the adipogenesis genes C/EBPα, C/EBPβ, and PPARγ [223]. In human primary skeletal muscle cells (hASCs) and in rats, HAdV-D36 infection upregulated transcription factors, including FoxO1 and PPARγ, as well as transcripts involved in fatty acid production, including PPARγ-regulated Cidec/FSP27, Acetyl-CoA carboxylase (ACC), SREBP-1c, SREBP-2, and 3-hydroxy-3-methylglutaryl-coenzyme A reductase (HMGR), in parallel reducing β-oxidation of fatty acids [[224], [225], [226]]. However, HAdV-D36 infection significantly reduced AMPK activity, which balances cellular energy homeostasis to promote catabolic pathways and inhibit anabolic pathways, uncoupling protein 3 (UCP3), and overall mitochondrial mass, which can lead to dysfunctional mitochondria energy regulation and β-oxidation [224,227,228]. Furthermore, HAdV-D36 increased glucose uptake and GLUT4 protein expression independently of insulin signaling in these cells, while decreasing IR substrate 1 (IRS1) tyrosine phosphorylation and IRS1 and IRS2-associated PI3K activities, meanwhile increasing Ras gene expression and protein expression [229]. In undifferentiated and differentiated hASCs, HAdV-D36 lastingly increased adiponectin abundance even in the absence of PPARγ, as well as glucose uptake and the expression of GLUT1 and GLUT4, suggesting that increased metabolism in adipose tissue is valuable to the HAdV-D36 infection [230].

Adiponectin is potently regulated by PI3K activity, and indeed *post hoc* screening of human sera showed that natural HAdV-D36 infections were correlated with greater adiponectin levels in humans [230,231]. Although current meta-analyses find that HAdV-D36 seropositivity is often associated but not always correlated with obesity, this nevertheless implies that obesity in humans may indeed be inducible by HAdV-D36 [[232], [233], [234], [235], [236], [237]]. Natural HAdV-D36 infections have been diagnosed by the presence of viral DNA in adipose tissue as well as neutralizing antibodies in sera, although it still needs to be determined whether adipose tissue hypertrophy is caused by HAdV-D36 infection or *vice versa* [21,238,239]. A US study found that HAdV-D36 infection was prevalent in approximately 30% of obese adults but only in 11% of non-obese adults, with the obesity-infection correlation also displaying paradoxically lower serum cholesterol and triglycerides, similar to animal models [23,217,239]. Similar results have shown the prevalence of HAdV-D36 infection and obesity varying across different countries, ranging from 65% to 6% [237,240]. Meta-analyses support the association or risk between HAdV-D36 infection and the development of obesity but seems to be more linked to the accumulation of subcutaneous fat rather than visceral fat [241,242].

HAdV-D36 infection has also been implicated in modulating the immune response, as shown in mesenchymal stem cells and adipocytes [218,243]. HAdV-D36 infection induced chronic inflammation by increasing macrophage chemoattractant protein 1 (MCP-1) and TNF levels in mice, activating nuclear factor kappa B (NF-κB), which releases pro-inflammatory cytokines and promotes macrophage infiltration into adipocytes as well as lipid accumulation [218]. MCP-1 plays a role in adipogenesis and recruiting monocytes and macrophages into adipose tissue, potentially contributing to insulin resistance and obesity development [244]. In humans, increased MCP-1 levels have been reported to correlate with anti-HAdV-D36 antibodies [218]. Furthermore, in 3T3-L1 cells and rats, HAdV-D36 infection can inhibit the gene expression of leptin, an adipocytokine or appetite hormone that among others regulates energy homeostasis and satiety, modulates immune function through the JAK-STAT pathway, and has a pro-inflammatory role while serving as a protective factor against infections [245,246]. Normally increased after lipid accumulation, a decrease in leptin due to HAdV-D36 infection may lead to increased lipid deposition, potentially increasing obesity [245].

E4orf1-D36 was first identified in 2008 as a causative agent that induces adipogenic differentiation and lipid accumulation in mouse 3T3-L1 and hASC cells, by blocking among other things IR-mediated tyrosine phosphorylation through IRS1 [22]. Since then, further transduction experiments with E4orf1-D36 *in vivo* revealed that the viral protein improves glycemic control and enhances glucose disposal independently of insulin, lowers non-fasting blood glucose levels, reduces serum levels of adiponectin, and increases body weight, meanwhile decreasing food intake and insulin levels without causing changes in liver or fat mass protein in a diabetic mouse model (*db/db*) [219,230,247,248]. E4orf1-D36 transduction also lowered blood glucose levels, increased glycemic control, and increased liver weight in diet-induced obese mice [219]. Molecularly, E4orf1-D36 transduction upregulated glycolysis-related transcripts in *db/db* mice and downregulated genes related to fatty acid synthesis; in diet-induced obese mice it upregulated glycolytic genes and other metabolic mRNAs in the liver.

In contrast, in wt mice E4orf1-D36 downregulated IR gene (INSR) expression independently of insulin expression, upregulated genes involved in glycolysis as well as the PDK4 gene, an inhibitor of the TCA cycle, downregulated genes involved in gluconeogenesis and glycogen formation, and downregulated G6PD, involved in the pentose phosphate pathway, and increased the prevalence of phospho-Akt and phospho-FoxO1, which corresponded to the decreased blood glucose levels that were measured [219]. Further *in vivo* and *in vitro* studies have reported similar effects of E4orf1-D36 and have found this protein to be necessary and sufficient to improve hyperglycemia and glucose uptake independently of endogenous insulin signaling and the IR, and to activate Ras and PI3K in a PBM-dependent manner *in vitro* and *in vivo* [[249], [250], [251], [252]]. Since E4orf1-D36 and HAdV-D36 infection metabolically remodel adipose tissue despite, for instance, a high fat diet, and do not require fat loss to improve glycemic control by circumventing insulin-IR-PI3K activation, this so-called insulin sparing action of E4orf1-D36 is being discussed as a potential therapeutic agent for improving hyperglycemia, attenuating hepatic steatosis, and treating insulin resistance [251,[253], [254], [255]]. For instance, novel research indicates that delivery of E4orf1 through nanoparticles to preadipocytes can restore glucose uptake to improve glycemic control in obese individuals and may provide a useful system for clinical applications [256].

## 9 HAdV triple agent 3: E4orf1 in HAdV-C5 – a multifunctional protein with conserved functions?

HAdV-C5 is one of the most extensively studied serotypes and exhibits a broad tropism, infecting a wide range of cell types in various tissues, including respiratory epithelium, ocular cells, and gastrointestinal epithelium [9,257]. The mechanisms underlying the specific functions of E4orf1-C5 are complex and involve interactions with several host cellular factors, often like, but also distinct from E4orf1-D9 and -D36.

For instance, evidence for the adipogenic effect of HAdV-C5 is complex and inconsistent. HAdV-C5 infection was reported to not induce lipid accumulation in mouse 3T3-L1 fibroblasts, although the data were never shown by the authors, while in Colo-320 cells HAdV-C5 infection was shown to have adipogenic potential [258,259]. In a recent study, expression of recombinant E4orf1-C5 in 3T3-L1 cells did indeed activate Akt and thus glucose uptake as well as FoxO1 exclusion from the nucleus, which is important for adipocyte differentiation. However, only E4orf1-C5 together with insulin was able to translocate GLUT4 to the plasma membrane, while E4orf1-C5 alone could redistribute GLUT1 [260,261]. Furthermore, HAdV-C5 infections were shown to induce adipogenic effects in mice and have been associated with obesity in humans in some studies, but this varies among different populations and age groups. Such discrepancies may be attributed to testing methods, geography, or other factors related to age and coinfections with other HAdV [202,239,262,263].

Some studies investigating HAdV-D36 have compared the metabolic actions of HAdV-C2. Although HAdV-C2 and -C5 are distinct virus species, their E4orf1 proteins share 100% homology (blastp [80]), indicating that the protein alone will have the same mode of action although the adenoviral infection context may differ. Usually, HAdV-C2 infections were used as an HAdV control, since in experiments such infections did not result in animal weight gain, adipocyte differentiation, lipid accumulation, decreased leptin levels, increased GLUT gene expression and glucose uptake, or overall improved glycemic control [221,222,226,229,230,245,264,265]. However, when investigated, phospho-Akt was mostly not activated in these studies, suggesting that the cellular systems tested were not adequate for full HAdV-C2 infection, despite being tested for HAdV-C2 DNA and mRNA expression [219,264].

Since we know that E4orf1-C5 can robustly activate Akt phosphorylation in human lung and mammary epithelial cells, as well as quiescent primary small airway epithelial cells (SAECs) it is feasible to suggest that E4orf1-C may have certain metabolic functions in the target tissue of HAdV-C species [92,266] (Göttig et al., JVI manuscript accepted). Indeed, one study investigated changes in cellular protein expression during HAdV-C2 infection in growth-arrested IMR-90 lung fibroblasts and found that proteins involved in glycolysis, purine and pyrimidine synthesis, as well as the glutathione pathway, serine glycine biosynthesis pathway and mannose metabolism were all upregulated during early infection [267]. By 12 hpi, enzymes of the fructose galactose metabolism and pentose phosphate pathway were upregulated [267]. Furthermore, the metabolism-associated transcription factors MYC, E2F1, ATF/CREB family, and NRF2 were increased, the latter also being attributed to regulating metabolism and inflammation [267,268]. In SAECs, as expected, E4orf1-C5 activated the PI3K-Akt-mTOR pathway, while an alternative E4 splicing product, E4orf4, shared a functionally redundant role in activating mTOR through the PI3K-Akt-mTOR effector p70^S6K^, independently of PI3K activity and downstream E2F transcription [266]. Since both proteins seem to cooperate to provide pro-survival cues by activating PI3K-Akt-mTOR and mTOR alone, it is thought that HAdV-C5 uses these mechanisms to induce efficient S-phase entry and viral replication [266].

The clearest data linking E4orf1-C5 to cellular metabolism was shown by Thai and colleagues, where E4orf1-C5 activated MYC in human epithelial cells, which seems to be related to the activity observed in HAdV-D36 and HAdV-D9 [134,200,223]. In two consecutive studies, transduction of E4orf1-C5 into epithelial cells was reported to contribute to establishing a cellular metabolic phenotype resembling cancer metabolism, where the upregulation of anabolic glucose metabolism, similar to the Warburg effect [199], cellular glycolysis and glutaminolysis was mediated through E4orf1 activation of MYC [200,201,269]. It was shown that E4orf1 alone binds MYC, which is strengthened by the interaction with E4orf6, enhancing MYC-mediated transcription of genes from both the nucleotide metabolism pathway, including pentose phosphate pathway transcripts, and genes from metabolic pathways, including HK2 and PFK1, but not GAPDH and LDHA, although MYC binding to GAPDH and LDHA gene loci was strengthened due to E4orf1 [200]. Thus, the metabolic changes induced by E4orf1 contributed to increased virus yield during infection and a point mutation in domain 2 of E4orf1 (D68A; corresponds to E4orf1-D9/D36 D65; Fig. 2B, grey boxes) as well as knockdown of MYC prevented the glycolytic metabolic changes and negatively impacted HAdV-C5 infection. However, E4orf1 did not decrease cellular respiration, as was detected in a HAdV-C5 ΔE4 mutant virus, indicating that further viral factors contribute to the metabolic reprogramming [200].

The metabolic pathway of glutaminolysis catabolizes glutamine to generate ATP and lactate. Cancer cells often exhibit an enhanced dependence on glutamine metabolism to support their growth and glutamine supplies carbon and nitrogen atoms for the synthesis of lipids, nucleotides, and specific amino acids, as well as facilitating the uptake of essential amino acids, which can signal downstream and activate mTOR [270,271]. Compared to HAdV-C5 wt, the HAdV-C5-E4orf1-D68A virus failed to induce glutaminolysis, or the upregulation of the glutamine transporters ASCT2 and LAT1, keeping intracellular concentrations of essential and non-essential amino acids low [201]. Furthermore, glutaminase levels were enhanced by wt but not mutant virus infection, thus promoting wt HAdV-C5 replication, and highlighting the importance of glutamine metabolism for viral replication [201]. Also, the hexosamine biosynthesis pathway was elevated in HAdV-C5 wt but not mutant infections, increasing UDP-GlcNAc production, which can modify metabolic enzymes involved in glycolysis [201].

Contrary to E4orf1-D36, E4orf1-C5 transduction *in vivo* does not improve glycemic control, enhance glucose disposal independently of insulin, lower (non-fasting) blood glucose levels, decrease food intake, cause changes in insulin, liver, body weight, or fat mass, nor cause significant changes in serum levels of adiponectin [219]. At the molecular level, E4orf1-C5 transduction *in vivo* does not significantly affect glycolytic genes, or MYC expression, but does upregulate some genes involved in lipid biosynthesis and induce phospho-Akt, and thus possibly increases the expression of certain metabolic genes, once again indicating that tissue-specificity of E4orf1-C5 expression may play a large role its metabolic activity [219].

Fundamental to tissue homeostasis is cell communication through gap junctions (GJ) and connexins (Cx), and an imbalance can lead to defective signal transduction and even cancer development [272]. In myocardial cells, Cx play a role in action potential propagation and immune responses, and HAdV-C5 infections can cause viral myocarditis and disrupt the Cx43 protein due to reduced GJA1 mRNA transcripts mediated by β-catenin activity [273]. Through PI3K-Akt-mTOR pathway activation, E4orf1-C5 induces β-catenin phosphorylation and thus transcriptional repression of the GJA1 target gene, resulting in loss of GJ function and altered GJ conductance [273]. Furthermore, E4orf1-C5 has been investigated as a biomolecular tool due to its PI3K-Akt-mTOR pathway activation, since it allows primary endothelial cells (PECs) to be cultured and purified without the need for exogenous cytokines or serum, which can alter the cells' angiogenic properties. Introducing E4orf1-C5 into PECs enhances their survival and preserves their ability to form new blood vessels *in vivo*. This provides researchers with a method to study and manipulate PECs for various applications, including investigating the role of vascular cells in organ-specific vascularization and tumor neo-angiogenesis [274]. These studies highlight once more, that the tissue specificity of HAdV-C5 infection/E4orf1-C5 expression may strongly determine the molecular disruptions mediated by the E4orf1-PI3K-Akt-mTOR pathway.

## 10 Biotechnological AdV vector applications and the role of E4orf1

Ever since it was first discovered that foreign DNA can be expressed in AdV genomes, recombinant AdV have been investigated as gene transfer vectors [275]. DNA cloning and recombination allows for gene delivery by AdV vectors to produce gene therapy candidates, used for instance in cancer treatment or immunostimulatory vaccines against infectious diseases. As vector candidates, most research has been conducted on HAdV types C2 and C5 [[276], [277], [278], [279]]. Conveniently, all AdV vectors have relatively high sequence packaging capacity, can be grown to high titer stocks, can infect non-dividing and dividing cells of different lineages, can efficiently transduce genes, exist only as episomes in cells, and can be genetically modified to be highly (vaccines) or poorly (gene therapy) immunogenic as replicating, attenuated, or replication-defective vectors [[280], [281], [282]]. Specific deletion of early expressed genes generates completely or selectively replication-defective vectors, which are still able to transduce and express therapeutic genes [283]. Nonetheless, high seroprevalence, pre-existing immunity, and neutralizing antibodies against common HAdV in humans can limit vector applications [284], and calls for the development of AdV vectors based on strains with little or no pre-existing immunity in human populations [285].

Since the construction, propagation, advantages/disadvantages, and application areas of such recombinant vectors have been reviewed extensively and recently [[286], [287], [288]], here, we only focus on the AdV proteins, their effects, and potential hazardous effects within AdV vector systems.

The first generation of AdV vectors were deleted for E1 often in combination with E3, thus eliminating viral transactivation and initiation of replication [289]. Second generation AdV vectors had further deletions in the E2 and/or E4 region [290], although at least E4orf3 or E4orf6 are necessary for vector growth in E1 trans-complementing cells, and complete removal of either E2 or E4 abrogates *de novo* replication [15,291,292]. Important to note is that gene expression from AdV vectors is reported to be leaky, suggesting that the absence of transactivating E1A does not ensure no AdV gene expression, especially for E2A, E4, pIX, hexon, and fiber [[293], [294], [295], [296], [297], [298], [299]]. In particular, inflammatory cytokine IL-6 and NF-κB have been shown to induce leaky expression of AdV vector promoters [300,301]. Although studies report that expression of the viral proteins are usually 10- to 100-fold lower than the transgene of interest [302,303], this may still lead to the production of adenoviral antigens that may trigger adaptive immune responses against AdV factors, or lead to a certain degree of cellular activity of the viral proteins, such as the E4 proteins, which could be relevant in cancer therapy [304].

The third class of non-replicative AdV are high capacity (HC; “gutted”) vectors, where all AdV genes are deleted except for the packaging sequence and the ITRs [305]. In the future, application of self-attenuating AdV, the removal of helper viruses using recombination strategies, such as the Cre-loxP system, or the use of helper virus-independent HC vectors may be promising approaches for genetic vector therapy [[305], [306], [307]].

Despite certain disadvantages of AdV vectors today, most vector-based clinical trials are conducted with AdV vectors [[308], [309], [310]], indicating that in the applied setting these systems are safe, adequately tolerable, immunogenic, and have therapeutic effect [[311], [312], [313], [314]]. Approximately 50% of gene therapy trials address cancer treatments where AdV vectors mediate transduction of tumor suppressors, suicide genes or tumor-specific promoters [309,315]. Furthermore, certain AdV vectors are selectively replication-competent, known as oncolytic viruses, designed to specifically target, conditionally replicate in, and destroy cancerous cells through lytic virus replication (reviewed in Ref. [316]. Modifications in one viral oncogene, E1A or E1B–55K, that interact with the cellular proteins Rb and p53, respectively, determine selective AdV replication in tumor cells that lack these cellular proteins. However, efforts are being made to increase tumor tropism by modulating the fiber gene, or to enhance the immune response towards tumors by deleting proteins of the E3 region, by co-expressing immune-stimulatory transgenes, or by including immune checkpoint inhibitors [[316], [317], [318]]. In 2006, the first oncolytic AdV, Oncorine (H101; Shanghai Sunway Biotech), based on the E1B–55K-deleted oncolytic ONYX-015 HAdV-C5 vector, was approved in China for clinical treatment of p53-deficient nasopharyngeal carcinoma in combination with chemotherapy [319]. Deleted for E1B–55K and E3, the tumor-specific replication is thought to be due to the lack of E1B–55K-mediated p53 deactivation as well as the loss of E1B–55K-mediated late viral RNA export [320]. Currently, Oncorine is being explored in other cancer therapies, and overall, more than 30 ongoing clinical trials are investigating oncolytic AdVs with several modifications in the viral genome to increase cancer cell selectivity [316,[321], [322], [323]](https://clinicaltrials.gov/, accessed on June 2023).

Notably, E4 region modifications are rarely utilized in oncolytic AdVs [316,324]. For instance, the ONYX-015 deletion mutant virus lacking the E1B–55K gene replicates less efficiently during cell cycle phase G1, which can predominate in tumors, limiting its clinical effectiveness [325,326]. The presence of E4orf1 within ONYX-015 reduces viral progeny production, inhibits viral late protein synthesis, and hampers tumor cell killing [327]. While the PI3K-Akt-mTOR pathway does not seem to be involved in E4orf1-mediated inhibition of late protein synthesis, it may still restrict virus-mediated cell killing, possibly modulating the efficiency of viral late mRNA translation and posttranslational processes, as well as potentially promoting PI3K-Akt-mediated pro-survival due to E4orf1 action [327]. Thus, E4orf1 and E1B–55K seem to have opposing effects, suggesting that rapid responses to environmental cues are balanced by these two proteins [327]. Moreover, phosphorylation of p70^S6K^ can occur independently of PI3K and Akt, with Rac1 and its interaction with Tiam1 playing a role [327]. Drugs targeting p70^S6K^ phosphorylation or the Tiam1-Rac1 interaction can enhance the cell-killing ability of ONYX-015, suggesting that not only knowledge about genetic and pharmacological strategies but also the role of E4orf1 within the AdV vectors can improve the efficacy of replicating oncolytic AdV for cancer treatment [327].

As our data have shown (Göttig et al., JVI manuscript accepted), specifically E4orf1 hinders E1B–55K-E4orf6-mediated E3 ubiquitin ligase degradation of the cellular target p53, displaying seemingly opposing HAdV replication strategies. However, this is counteracted by p53 itself, which possibly initially hinders a hyperactivating PI3K-Akt-mTOR signaling axis, while concurrently being available for degradation by the E3 ubiquitin ligase complex. Furthermore, prolonged activation of the PI3K-Akt-mTOR pathway may, under the correct circumstances and sufficient expression levels, be unchecked in a p53-deficient cancer cell, implying that these pro-survival cues from E4orf1 in oncolytic AdV need to be investigated in the future.

HAdV-C5 transduction efficiency in human solid tumors is often insufficient, calling for better transducible systems. One study determined that HAdV-D9 showed better virus-spread ability and progeny release compared to other HAdV serotypes from A-G. It was reported that the fiber protein of HAdV-D9 increased its tropism for infection by attaching to target cells independently of the HAdV-common coxsackievirus and adenovirus receptor (CAR), killing both CAR-positive and CAR-negative cancer cells, suggesting better therapeutic efficacy in solid tumors than with HAdV-C5 [328]. Despite the study being limited to testing virus spread in *in vitro* plaque assays and *in vitro* cell killing of cancer cell lines, it is also feasible that the highly oncogenic potential of HAdV-D9 and E4orf1 expression may have additive tumorigenic effects due to the complexity of the deregulated cancer environment.

As prophylactic vaccines, AdV vectors can induce strong immunogenicity when a transduced antigenic protein is fully expressed to trigger adaptive immunity [281,329]. Since the emergence of Severe Acute Respiratory Syndrome Coronavirus 2 (SARS-CoV-2) at the end of 2019, global efforts have achieved the development of effective vaccines, and overall five different AdV vector vaccines were initially licensed (reviewed in Ref. [330]). For all AdV vector vaccines against SARS-CoV-2, deletion of E1 genes allowed the incorporation of genes encoding the SARS-CoV-2 spike protein. The replication incompetent AdV vector vaccine is injected into the recipient, triggering humoral and cellular immune responses to develop neutralizing antibodies against the cell-transcribed and translated SARS-CoV-2 spike [331]. Although these vaccines were classified as effective and safe, expression of AdV genes was detectable in human lung fibroblast MRC5 cells and human lung epithelial A549 cells, which may lead to increased immune responses upon vaccination, but also have so far undefined consequences due adenoviral protein expression [303,[332], [333], [334], [335], [336]].

Since especially the E4 proteins are associated with a certain degree of oncogenic potential, studies investigating this remain to be conducted [42]. Furthermore, a prophylactic UV-attenuated HAdV-D36 vaccine has been discussed, at least experimentally, to be protective against HAdV-D36-induced obesity and inflammation. In mice, the control group had 17% greater body weight and 20% more epididymal fat than vaccinated mice, which actually had decreased serum levels of pro-inflammatory cytokines, and immune cells infiltrating fat tissue, suggesting that this specific vaccination strategy was protective against HAdV-D36-increased body weight, fat deposition, and inflammatory states [[337], [338], [339]].

Eliciting a robust immune response is critical to an effective vaccination strategy. Interestingly, E1B–55K and the E4 genes reduce the innate immune response of HAdV-infected cells [[340], [341], [342]]. Thus, deleting E1B–55K and the E4 region in an HIV-1-protective HAdV-C5 vector promoted higher levels of HIV-1 envelope-specific binding antibodies in immunized mice and rhesus macaques than either a deleted E3 or E1B–55K vector, suggesting that the E4 proteins within a HAdV vector limit the immune response [25]. Recently, Sangare and co-authors identified E4orf1 as a co-factor with E1B–55K that limited the immune response, since rhesus macaques immunized by an E1B–55K- and E4orf1-deleted HAdV-C5 vector produced higher levels of HIV-envelope-specific interferon γ-producing memory T-cells, higher titers of specific IgG1 binding antibody, and antibodies that were able to mediate antibody-dependent cellular cytotoxicity with greater killing capacity than the original deleted E1B–55K vector [25]. Since the PI3K-Akt-mTOR pathway also mediates immune responses through activating the NF-κB-mediated transcription of cytokine genes that are important for stimulating innate and adaptive parts of the immune system, E4orf1 activation of PI3K may also induce NF-κB and seems to be involved in HAdV-C5 vector system immune response signaling [25,[343], [344], [345]]. Although it remains to be investigated to what degree E4orf1 is “active” within AdV vectors, these studies indicate that the inclusion of the E4orf1 coding region clearly affects immunogenicity and oncolysis of these vectors [25,327].

## 11 Conclusions and future perspectives

In summary, E4orf1-D9, -D36, and -C5 have specific activating functions mostly mediated through the PI3K-Akt-mTOR pathway (Fig. 3). However, the induction of downstream signaling pathways can be numerous, since cell type, cellular environment, and HAdV type play a determining role, highlighting that HAdV infection and E4orf1 expression can lead to specialized pathogeneses depending on the infected or transduced tissue and experimental model. Therefore, to what extent E4orf1 expression and functional activity within infection and vector systems might be active or even harmful to the host remains an open question. Equally, it is unclear whether certain E4orf1 functions, such as oncogenesis or metabolism activation, will prove to be beneficial or detrimental tools for treating insulin resistance or other metabolic disorders, protecting against infectious weight gain, administering gene therapy, or treating cancer with oncolytic viruses, in the presence of HAdV oncogenes such as E4orf1.

In this review, we have explored the gene composition, protein domains and structure, as well as functions and significance of E4orf1, depending on the HAdV type and the target tissue. We have discussed its role in viral replication, modulation of host immune responses, and activation of cellular signaling networks, including PI3K-Akt-mTOR, Raf-MEK-ERK, Ras, insulin, EGF, MYC, and NF-κB signaling. Specifically, we have highlighted its involvement in oncogenesis in HAdV-D9, in adipogenesis and metabolic modulation in HAdV-D36-associated obesity, and multifunctional metabolic, oncogenic, and immunostimulatory functions in HAdV-C5. Several key findings have emerged:

• E4orf1 activates PI3K-Akt-mTOR and interacts with PI3K, facilitated by the PDZ protein Dlg1 and Ras activation, which is conserved between HAdV serotypes.
• The PI3K-Akt-mTOR pathway is more favored than Raf-MEK-ERK activation, which are both Ras-dependent.
• MYC activation and interaction with E4orf1 ensures efficient transcription of metabolic MYC-target genes; MYC activation is conserved between HAdV serotypes.
• E4orf1 exhibits serotype-specialized functions and interactions, contributing to the distinct pathology of different HAdV serotypes.
• E4orf1-D9 is an oncogene in non-allogenic systems *in vitro* and *in vivo*, inducing mammary tumors in rats; metabolic reprogramming can occur.
• E4orf1-D36 activates adipogenesis and metabolic glucose and lipid regulation, potentially contributing to obesity-related disorders in humans.
• E4orf1-C5 is a multifunctional protein that can be tumorigenic in non-allogenic *in vitro* systems, and reprograms cellular glucose and lipid metabolism *in vitro* and *in vivo*, but seems to have diminished metabolism reprograming functions *in vivo.*
• E4orf1-C5 inhibits the oncolytic potential of E1B–55K-deleted HAdV vectors.
• E4orf1-C5 limits the immune response in E1B–55K-deleted HAdV vector vaccines.

Exploring the impact of E4orf1 in other AdV serotypes beyond HAdV-C5, HAdV-D9, and HAdV-D36 will be important for comprehensively understanding E4orf1's role in AdV pathogenesis. Investigating the functions and interactions of E4orf1 in additional serotypes may uncover novel or conserved mechanisms and shed light on the broader significance of this protein. Additionally, further research is needed to understand the implications of E4orf1 in obesity-related disorders. Elucidating the molecular mechanisms *in vivo* by which E4orf1 influences adipogenesis, metabolic regulation, and inflammation in HAdV-D36 infection will aid in better understanding obesity, insulin resistance, and associated comorbidities. Modulating E4orf1-mediated adipogenesis, metabolic pathway activation or pro-survival functions in model systems could be a powerful tool for identifying potential therapeutic targets for obesity-associated diseases.

In conclusion, E4orf1 plays a pivotal role in AdV infection and associated diseases. The multifunctional nature of E4orf1 due to its involvement in a multifunctional network of protein signaling highlights its importance within HAdV infections and target cells. Further research into the specific mechanisms and interactions of E4orf1 will enhance our understanding of HAdV pathogenesis and pave the way for the development of novel therapeutic strategies and improved gene transfer or vaccination vectors.

## CRediT authorship contribution statement

**Lilian Göttig:** Writing – review & editing, Writing – original draft, Visualization, Conceptualization. **Sabrina Schreiner:** Writing – review & editing, Writing – original draft, Visualization, Conceptualization.

## Declaration of competing interest

The authors declare that they have no known competing financial interests or personal relationships that could have appeared to influence the work reported in this paper.

## Data Availability

No data was used for the research described in the article.
